# Are Different Diseases in Old Age Connected With Different Attitudes Toward Own Aging and Subjective Age?

**DOI:** 10.1093/geroni/igaa057.1972

**Published:** 2020-12-16

**Authors:** Anton Schönstein, Hans-Werner Wahl, Michael Denkinger, Dhayana Dallmeier, Dietrich Rothenbacher, Jochen Klenk, Anke Bahrmann

**Affiliations:** 1 University of Heidelberg, Heidelberg, Baden-Wurttemberg, Germany; 2 Agaplesion Bethesda Clinic, Ulm, Baden-Wurttemberg, Germany; 3 University of Ulm, AGAPLESION Bethesda Clinic, Geriatric Center Ulm/Alb-Donau, Ulm, Baden-Wurttemberg, Germany; 4 Ulm University, Ulm, Baden-Wurttemberg, Germany; 5 Institute of Epidemiology and Medical Biometry, Ulm University, Ulm, Baden-Wurttemberg, Germany; 6 Heidelberg University Hospital, Heidelberg, Baden-Wurttemberg, Germany

## Abstract

Subjective views on aging (VoA; e.g., subjective age, attitude toward own aging “ATOA”) are regarded as important biopsychosocial markers of aging but their antecedents are not entirely clear. Besides general risk factors (depression, cognition, activities of daily living), we compared multiple disease groups to establish connections between specific morbidities and risk for negative VoA. Data was drawn from the ActiFE-Ulm study for which a representative sample of community-dwelling older people (65-90 years) was recruited. Follow-ups were conducted 7.7 years (median) after recruitment (T2; N=526). Self-reported depression at T1 was the strongest general risk-factor for negative VoA at follow-up (both subjective age and ATOA). Back pain predicted negative ATOA, whereas rheumatism was associated to both negative ATOA and older subjective age. We conclude that diseases are differentially associated with VoA. Further, mental health problems such as depression seem to be of higher importance for VoA as compared to other factors.

